# Cross-Clade Memory Immunity in Adults Following SARS-CoV-1 Infection in 2003

**DOI:** 10.1001/jamanetworkopen.2022.47723

**Published:** 2022-12-20

**Authors:** Rita W. Y. Ng, Siaw S. Boon, Zigui Chen, Wendy C. S. Ho, Kitty S. C. Fung, Barry K. C. Wong, Apple C. M. Yeung, Martin C. S. Wong, Paul K. S. Chan

**Affiliations:** 1 Department of Microbiology, Faculty of Medicine, Chinese University of Hong Kong, Hong Kong; 2 Department of Pathology, United Christian Hospital, Hong Kong; 3 JC School of Public Health and Primary Care, Chinese University of Hong Kong, Hong Kong; 4 Stanley Ho Centre for Emerging Infectious Diseases, Faulty of Medicine, Chinese University of Hong Kong, Hong Kong

## Abstract

**Importance:**

Knowledge of the longevity and breath of immune response to coronavirus infection is crucial for the development of next-generation vaccines to control the COVID-19 pandemic.

**Objectives:**

To determine the profile of SARS-CoV-2 antibodies among persons infected with the closely related virus, SARS-CoV-1, in 2003 (SARS03 survivors) and to characterize their antibody response soon after the first and second doses of COVID-19 vaccines.

**Design, Setting, and Participants:**

This prospective cohort study examined SARS-CoV-2 antibodies among SARS03 survivors compared with sex- and age-matched infection-naive controls. Participants received the COVID-19 vaccines between March 1 and September 30, 2021.

**Interventions:**

One of the 2 COVID-19 vaccines (inactivated [CoronaVac] or messenger RNA [BNT162b2]) available in Hong Kong. Two doses were given according to the recommended schedule. The vaccine type administered was known to both participants and observers.

**Main Outcomes and Measures:**

SARS-CoV-2 antibodies were measured prevaccination, 7 days after the first dose, and 14 days after the second dose.

**Results:**

Eighteen SARS03 adult survivors (15 women and 3 men; median age, 46.5 [IQR, 40.0-54.3] years) underwent prevaccination serologic examination. The vast majority retained a detectable level of antibodies that cross-reacted with SARS-CoV-2 (16 of 18 [88.9%] with nucleocapsid protein antibodies and 17 of 18 [94.4%] with receptor-binding domain of spike protein antibodies); a substantial proportion (11 of 18 [61.1%]) had detectable cross-neutralizing antibodies. Twelve SARS03 adult survivors (10 women and 2 men) underwent postvaccination serologic examination. At 7 days after the first dose of vaccine, SARS03 survivors mounted significantly higher levels of neutralizing antibodies compared with controls (median inhibition: 89.5% [IQR, 77.1%-93.7%] vs 13.9% [IQR, 11.8%-16.1%] for BNT162b2; 64.9% [IQR, 60.8%-69.5%] vs 13.4% [IQR, 9.5%-16.8%] for CoronaVac; *P* < .001 for both). At 14 days after the second dose, SARS03 survivors generated a broader antibody response with significantly higher levels of neutralizing antibodies against variants of concern compared with controls (eg, median inhibition against Omicron variant, 52.1% [IQR, 35.8%-66.0%] vs 14.7% [IQR, 2.5%-20.7%]; *P* < .001).

**Conclusions and Relevance:**

The findings of this prospective cohort study suggest that infection with SARS-CoV-1 was associated with detectable levels of antibodies that cross-react and cross-neutralize SARS-CoV-2, which belongs to a distinct clade under the same subgenus *Sarbecovirus*. These findings support the development of broadly protective vaccines to cover sarbecoviruses that caused 2 devastating zoonotic outbreaks in humans over the last 2 decades.

## Key Points

**Question:**

Does SARS-CoV-1 infection confer long-lasting memory immunity against the closely related SARS-CoV-2?

**Findings:**

This cohort study of 12 participants with SARS-CoV-1 infection in 2003 showed robust antibody response at 7 days after receiving 1 dose of either inactivated or messenger RNA COVID-19 vaccine. After 2 doses, those with past SARS-CoV-1 infection developed significantly higher levels of neutralizing antibodies against wild-type SARS-CoV-2 and variants of concern compared with 40 sex- and age-matched SARS-CoV-1–naive controls.

**Meaning:**

These findings suggest that cross-clade memory immunity persists long after natural infection with SARS-CoV-1, supporting the development of vaccines with a broad coverage to control pandemics due to coronaviruses.

## Teaser

This cohort study examines whether infection with SARS-CoV-1 in 2003 is associated with short-term antibody response to an inactivated or a messenger RNA–based COVID-19 vaccine among adults in Hong Kong.

## Introduction

Among the 7 human coronaviruses known to date, 3 have been identified as the cause of severe acute respiratory syndrome, including the severe acute respiratory syndrome coronavirus (SARS-CoV-1) that emerged in late 2002,^1^ the Middle East respiratory syndrome coronavirus (MERS-CoV) that emerged in 2012,^2^ and SARS-CoV-2 that emerged in late 2019.^3,4^ The other 4 coronaviruses (OC43, HKU1, 229E, and NL63) are endemic and mainly cause the common cold.

SARS-CoV-1 and SARS-CoV-2 share a most common ancestor within the subgenus *Sarbecovirus*, whereas MERS-CoV belongs to another subgenus *Merbecovirus*.^5^ Despite sharing a high degree (79.6%) of genome sequence identity and belonging to the same virus species, SARS-CoV-1 and SARS-CoV-2 are antigenically placed into 2 distinct phylogenetic clades.^3^ Studies on cross-neutralization between SARS-CoV-1 and SARS-CoV-2 are limited and remain inconclusive.^3,6,7,8^

Knowledge of cross-reactivity and cross-immunity among coronaviruses is important for interpreting seroprevalence studies and developing next-generation pancoronavirus vaccines that cover emerging variants. We conducted the current study to examine whether infection with SARS-CoV-1 in 2003 (SARS03) influences the short-term antibody response to vaccination with the Vero cell–based inactivated vaccine (CoronaVac) or with a messenger RNA (mRNA)–based vaccine (BNT162b2).

## Methods

The study was approved by the Joint Chinese University of Hong Kong–New Territories East Cluster Clinical Research Ethics Committee. Written informed consent was obtained from all participants. We followed the Strengthening the Reporting of Observational Studies in Epidemiology (STROBE) reporting guideline.

### Study Participants

We assumed that 80% of individuals who had survived previous laboratory-confirmed SARS03 and 40% of controls would produce detectable cross-reacting antibodies after vaccination. Twenty participants in each group would be required to achieve a power of 80% and a confidence level of 95% as determined using a sample size calculator (Select Statistical Services Ltd).^9^ We therefore targeted to recruit 20 individuals in the SARS03 group and 20 controls for each vaccine type.

We recruited SARS03 survivors by advertising this study through the health care workers in a few major hospitals where a SARS-CoV-1 outbreak had occurred. Health care workers and their network friends and relatives were welcome. Participants were reviewed for their likelihood of having had SARS-CoV-2 based on having compatible symptoms since the beginning of the pandemic, being quarantined, or being classified as a close contact of infected persons. SARS03 survivors without a history of suspected COVID-19 were classified as SARS-CoV-2 naive and were all enrolled to this study without further selection. We did not use serology to determine the status of SARS-CoV-2 exposure among SARS03 survivors, because we hypothesized that they may carry cross-reacting antibodies. At the time of this study, Hong Kong still adopted a stringent containment strategy with intense contact tracing, quarantining, and compulsory testing of individuals with suspected infection; the prevalence of SARS-CoV-2 was estimated to be less than 0.45%.^10^

A baseline blood sample was taken from participants, and they were then allowed to decide whether to receive a SARS-CoV-2 vaccine and given a free choice between the 2 types available in Hong Kong. These included the inactivated CoronaVac and the mRNA-based BNT162b2 vaccines. Those who chose vaccination underwent blood sampling 7 days after the first dose and 14 days after the second dose of vaccine.

Healthy persons naive to SARS-CoV-1 and SARS-CoV-2 infections were invited to participate in this study as controls. We advertised this study through media and social networks, and individuals who had participated in a previous study by Boon et al^10^ on seroprevalence of SARS-CoV-2 infection were also invited. Persons without a history of SARS-CoV-1 infection in 2003 were regarded as naive to SARS-CoV-1, whereas SARS-CoV-2 status was confirmed by serology in addition to those criteria as described for SARS03 survivors. Interested participants were asked about their intended choice of vaccine, and 20 participants for each vaccine type were enrolled on a first-come, first-served basis. Controls underwent blood sampling within the same time frame as SARS03 survivors. All participants completed vaccination between March 1 and September 30, 2021, when the prevalence of COVID-19 was estimated to be very low, at less than 0.45%.^10^

### SARS-CoV-2 Antibody Profile

Prevaccination blood samples were tested for antibodies against the nucleocapsid protein (anti-N) and the receptor-binding domain of spike protein (anti-RBD) of SARS-CoV-2 using electrochemiluminescence immunoassays (Elecsys anti–SARS-CoV-2 and anti–SARS-CoV-2 S; Roche Diagnostics GmbH). Additional testing was performed for neutralizing antibodies against the wild-type SARS-CoV-2 using a surrogate virus neutralization test (sVNT) kit (GenScript USA Inc).

Blood samples taken 7 days after the first dose of vaccine were tested for anti–SARS-CoV-2 neutralizing antibodies to indicate memory humoral immune response. Samples taken 14 days after the second dose of vaccine were tested for neutralizing antibodies against wild-type SARS-CoV-2 and variants of concern to determine the breadth of cross-neutralization.

### Statistical Analysis

Sex distribution between groups was compared via a Fisher exact test using StatCalc (Epi Info, version 7.2.5.0 [US Centers for Disease Control and Prevention]). Age and antibody levels between groups were compared using the Wilcoxon rank sum test in the R Stats Package, version 4.2.1 (R Foundation for Statistical Computing). Two-tailed *P* values of less than .05 were regarded as statistically significant.

## Results

**Figure 1.**
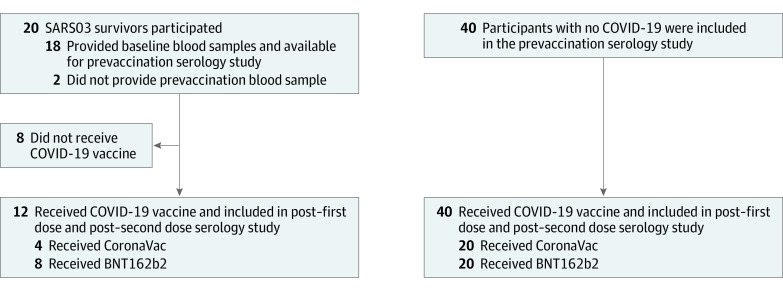
Study Flow Diagram Participants elected to receive a Vero cell–based inactivated vaccine (CoronaVac) or a messenger RNA–based vaccine (BNT162b2). SARS03 indicates SARS-CoV-1 infection in 2003.

Altogether, 20 SARS03 survivors and 40 controls were enrolled in this study (Figure 1). Five SARS03 survivors were taking medications for chronic medical problems, including 2 with hypertension, 1 with hypercholesterolemia, 1 with chronic hepatitis B infection, and 1 with hypertension, type 2 diabetes, and depression. Three controls were taking medications for hypertension, and 1 was taking antidepressants. None of the SARS03 survivors and controls were known to be immunosuppressed. Data on the severity of and treatment received for SARS-CoV-1 infection in 2003 were not available.

### SARS03 Survivor Prevaccination Serologic Profile

**Figure 2.**
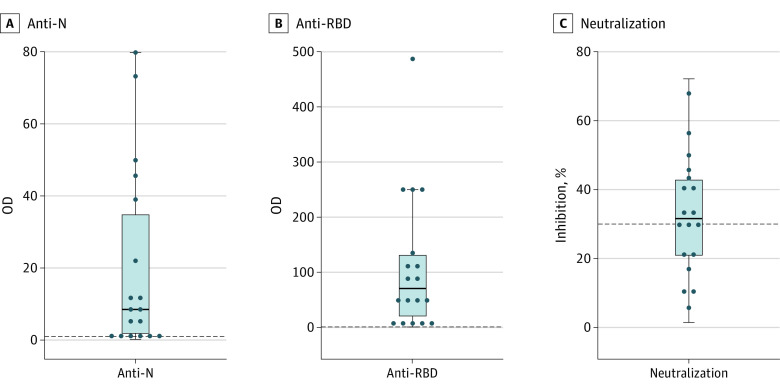
Box Plots of Anti–SARS-CoV-2 Antibodies Before Vaccination in Individuals With SARS-CoV-1 Infection in 2003 Antibodies to nucleocapsid protein (anti-N) and to receptor-binding domain of spike protein (anti-RBD) were measured using anti–SARS-CoV-2 and anti–SARS-CoV-2 S electrochemiluminescence immunoassays (Elecsys; Roche Diagnostics GmbH), and neutralizing antibodies were measured using the SARS-CoV-2 surrogate virus neutralization test kit (wild-type) (GenScript USA Inc). The dotted line represents cutoff optical density (OD; 1 for anti-N and 0.8 for anti-RBD) or percentage inhibition (30% for neutralizing) to classify positive samples according to the manufacturer’s instructions. The top and bottom of each box indicate the 75th and 25th percentiles, and the horizontal line inside each box indicates the 50th percentile.

Eighteen SARS03 survivors (15 women and 3 men; median age, 46.5 [IQR, 40.0-54.3] years) provided baseline blood samples. Their blood samples were collected at a median of 45 (IQR, 27-105) days before the first dose of vaccine. The proportions of samples positive for SARS-CoV-2 anti-N and anti-RBD antibodies were 16 of 18 (88.9%) and 17 of 18 (94.4%), respectively. The proportion of samples positive for neutralizing antibodies against wild-type SARS-CoV-2 was 11 of 18 (61.1%) when a 30% cutoff was used (Figure 2). The prevaccination blood samples of controls were collected at a median of 4 (IQR, 2-6) days before the first dose of vaccine, and all tested negative for SARS-CoV-2 antibodies.

Altogether, 12 SARS03 survivors received 2 doses of COVID-19 vaccine, whereas the others refused vaccination for personal reasons. Since the duration between enrollment and vaccination varies, the history of COVID-19 was reviewed again at postvaccination blood sampling. None of the SARS03 survivors were suspected to have COVID-19 before vaccination. Among the 12 who received the vaccine, 4 (3 women and 1 man; median age, 52.5 [IQR, 41.8-57.3] years) received CoronaVac; 8 (7 women and 1 man; median age, 41.5 [IQR, 40.0-51.3] years) received BNT162b2. A total of 20 controls received CoronaVac (16 women and 4 men; median age, 50.0 [IQR, 42.0-55.8] years); another 20 controls received BNT162b2 (17 women and 3 men; median age 45.0 [IQR, 41.0-47.8] years). There were no significant differences in sex and age distribution between SARS03 survivors and controls among CoronaVac recipients (ratio of women to men, 3:1 vs 4:1 [*P* > .99 by 2-tailed Fisher exact test]; median age, 52.5 [IQR, 41.8-57.3] vs 50.0 [IQR, 42.0-55.8] years; *P* = .64 by Wilcoxon rank sum test) and BNT162b2 recipients (ratio of women to men, 7:1 vs 5.6:1 [*P* > .99 by 2-tailed Fisher exact test]; median age, 41.5 [IQR, 40.0-51.3] vs 45.0 [IQR, 41.0-47.8] years; *P* = .34 by Wilcoxon rank sum test).

**Figure 3.**
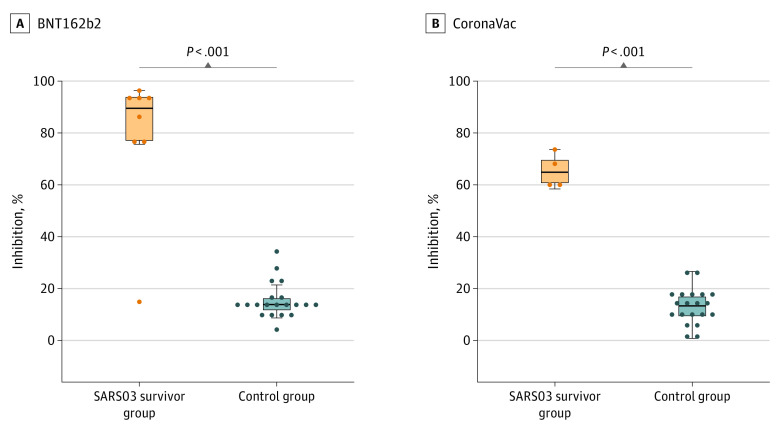
Box Plots of Early Antibody Response After the First Dose of Vaccine All participants received a Vero cell–based inactivated vaccine (CoronaVac) or a messenger RNA–based vaccine (BNT162b2). All samples were collected a median of 7 (range, 6-10) days after the first dose of vaccine, except for 1 individual with SARS-CoV-1 infection in 2003 (SARS03) whose sample was collected 18 days after the first dose of the CoronaVac vaccine. Neutralizing antibodies were measured by the SARS-CoV-2 surrogate virus neutralization test kit (wild-type) (GenScript USA Inc). The top and bottom of each box indicate the 75th and 25th percentiles, and the horizontal line inside each box indicates the 50th percentile. *P* values were obtained by Wilcoxon rank sum test.

Figure 3 shows the levels of SARS-CoV-2 antibodies in SARS03 survivors and controls measured from samples collected at a median of 7 (range, 6-10) days after the first dose of COVID-19 vaccine. SARS03 survivors mounted a significantly higher level of neutralizing antibodies compared with controls after receiving 1 dose of either BNT162b2 (median inhibition, 89.5% [IQR, 77.1%-93.7%] vs 13.9% [IQR, 11.8%-16.1%]; *P* < .001 by Wilcoxon rank sum test) or CoronaVac (median inhibition, 64.9% [IQR: 60.8%-69.5%] vs 13.4% [IQR, 9.5%-16.8%]; *P* < .001 by Wilcoxon rank sum test).

**Figure 4.**
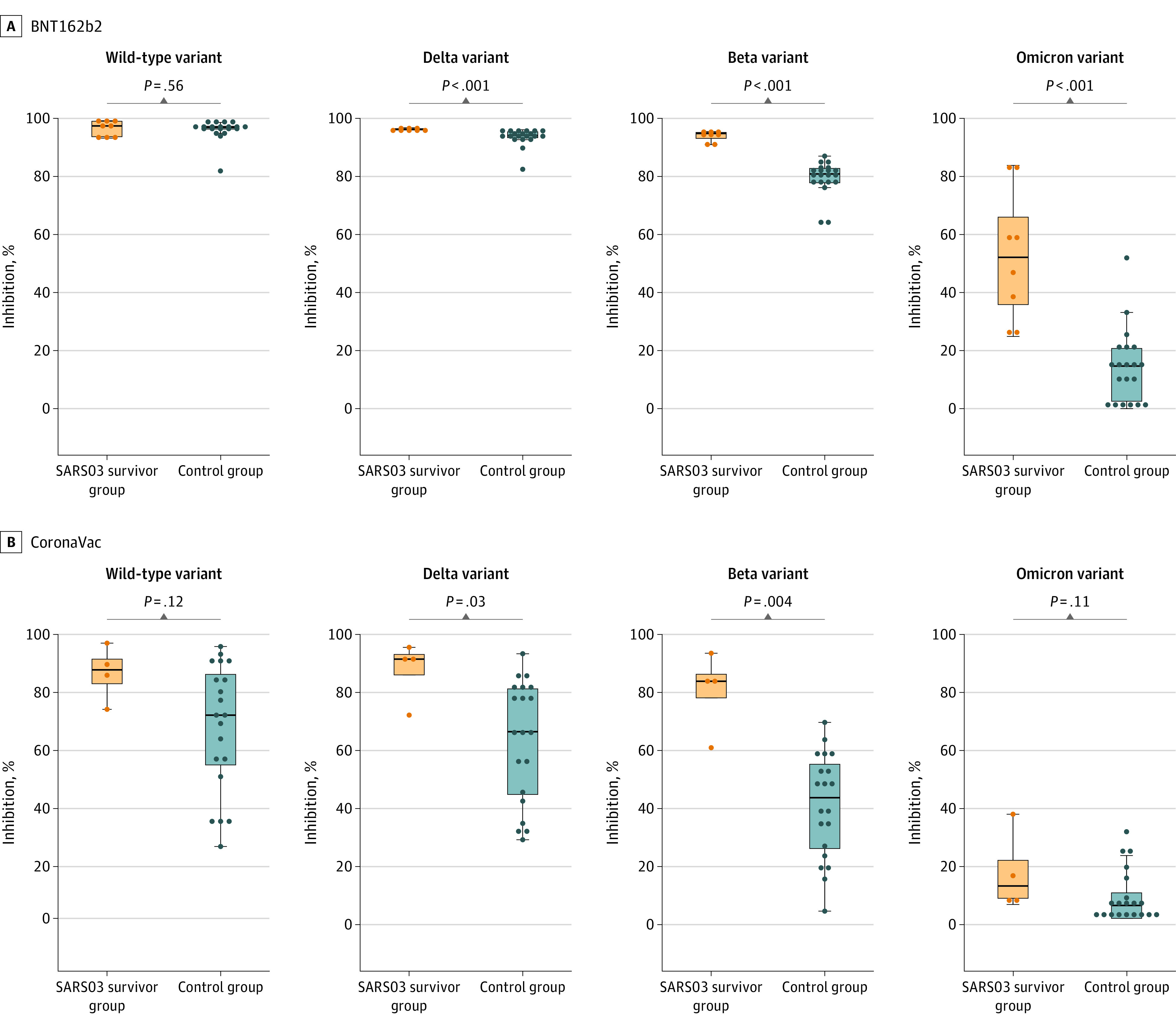
Box Plots of Antibody Response Against Wild-Type SARS-CoV-2 and Variants of Concern After 2 Doses of Vaccine All participants received a Vero cell–based inactivated vaccine (CoronaVac) or a messenger RNA–based vaccine (BNT162b2). All samples were collected a median of 14 (range, 13-19) days after the second dose of vaccine. Neutralizing antibodies were measured by the SARS-CoV-2 surrogate virus neutralization test kit (GenScript USA Inc) incorporated with the recombinant receptor-binding domain (RBD) fragment of the wild-type variant or variants of concern as indicated. The upper panel shows BNT162b2 and the lower panel shows CoronaVac. The top and bottom of each box indicate the 75th and 25th percentiles, and the horizontal line inside each box indicates the 50th percentile. *P* values were obtained by Wilcoxon rank sum test.

The second dose was administered at a median of 21 (IRQ, 17-33) days and 28 (IRQ, 28-34) days after the first dose of BNT162b2 or CoronaVac, respectively. Figure 4 shows the levels of SARS-CoV-2 antibodies in samples collected at a median of 14 (range, 13-19) days after receiving the second dose of COVID-19 vaccine. Among BNT162b2 recipients, both SARS03 survivors and controls mounted a high level of neutralizing antibodies against wild-type SARS-CoV-2 (median inhibition, 97.4% [IQR, 93.7%-99.0%] vs 96.9% [IQR, 96.3%-97.3%]; *P* = .56 by Wilcoxon rank sum test). The antibody levels against all variants of concern examined in this study were significantly higher among SARS03 survivors compared with controls (median inhibition for the Delta variant, 96.2% [IQR, 96.1%-96.5%] vs 94.1% [IQR, 93.4%-95.4%]; for the Beta variant, 94.8% [IQR, 93.1%-95.2%] vs 80.9% [IQR, 77.8%-82.8%]; for the Omicron variant, 52.1% [IQR, 35.8%-66.0%] vs 14.7% [IQR, 2.5%-20.7%]; *P* < .001 by Wilcoxon rank sum test for all).

The pattern of antibody levels observed from CoronaVac recipients was similar to that from BNT162b2 recipients (Figure 4). While there was no statistically significant difference in neutralizing antibody levels against wild-type SARS-CoV-2 (87.5% [IQR, 82.6%-91.3%] vs 71.6% [IQR, 54.0%-85.9%]; *P* = .11 by Wilcoxon rank sum test), the levels were significantly higher among SARS03 survivors compared with controls for the Delta variant (91.3% [IQR, 85.7%-92.9%] vs 65.7% [IQR, 43.6%-80.8%]; *P* = .03 by Wilcoxon rank sum test) and Beta variant (83.5% [IQR, 77.6%-86.0%] vs 42.5% [IQR, 24.6%-54.3%]; *P* = .004 by Wilcoxon rank sum test). The antibody level against the Omicron variant appeared higher for SARS03 survivors (11.4% [IQR, 7.1%-20.4%] vs 4.6% [IQR, 0.0%-9.0%]; *P* = .11 by Wilcoxon rank sum test).

## Discussion

The current pandemic virus, SARS-CoV-2, was first detected in China in late December 2019.^3^ While outbreaks from other parts of the world were soon detected, their mortality appeared much higher than that reported from China.^11,12,13,14,15^ Reasons for the difference in mortality could be complex, involving viral virulence, host health condition, containment measures, and other epidemiological factors. Of note, in late 2002, SARS-CoV-1 emerged and widely spread in China.^16^ We are intrigued to know whether such exposure to the closely related virus could have conferred a certain degree of immunity against newly emerged SARS-CoV-2.

In this study, we did not use SARS-CoV-2 serology to define COVID-19 exposure among SARS03 survivors, because our hypothesis was that persons infected with SARS-CoV-1 retain antibodies that cross-react with the closely related virus, SARS-CoV-2. In fact, our findings support this hypothesis. Our finding that a high proportion of SARS03 survivors tested positive for SARS-CoV-2 antibodies at prevaccination could not be explained by natural infection, since the prevalence of COVID-19 in Hong Kong was very low (<0.45%) at the time.^10^ Thus, our observed results were likely due to cross-reactivity, which should be considered when interpreting seroprevalence data in places where SARS-CoV-1 had circulated. Furthermore, based on a surrogate neutralization test, we found neutralizing antibodies against SARS-CoV-2 in most SARS03 survivors, which is in contrast to the negative findings from a previous study using a live virus–based neutralization test.^6^ The difference in sensitivity between the methods used could be a reason contributing to the discrepant observation.

We documented that despite the remote history of exposure to SARS-CoV-1, administration of 1 dose of either mRNA or inactivated SARS-CoV-2 vaccine resulted in detection of a robust level of neutralizing antibodies as early as 7 days after vaccination. This indicates a cross-reacting memory immune response on challenge with a closely related virus, SARS-CoV-2. Furthermore, this booster response generated antibodies broadly reactive to variants of concern. Our observations are in line with previous ex vivo studies that demonstrated a long-lasting T- and B-cell immunity cross-recognizing SARS-CoV-2 persists in SARS survivors.^17,18^ Furthermore, Tan et al^19^ also reported that SARS-CoV-1 survivors, on being immunized with mRNA COVID-19 vaccine, developed a broad spectrum of antibodies that cross-neutralized sarbecoviruses identified in bats and pangolins.

### Limitations

In this study, we used a surrogate method (sVNT) to measure neutralizing antibody response. There are limitations of sVNT. First, sVNT assays measure anti-RBD antibodies and quantify the binding affinity to infer neutralizing capacity; therefore, neutralizing antibodies against other domains of the S protein are missed.^20^ It has been shown that sVNT assays lack sensitivity to detect low titers of neutralizing antibodies.^21^ Therefore, those SARS03 survivors with undetectable sVNT antibodies at prevaccination could have a low level of neutralizing antibodies. Second, sVNT results show only partial linearity with the criterion standard plaque reduction neutralization test of 50% titers.^21^ Therefore, the differences in sVNT results observed in this study may not be directly proportional to the protective effect of vaccination.

Another limitation of our study is the number of SARS03 survivors available, especially those who opted for inactivated COVID-19 vaccine, was less than our targeted sample size. Furthermore, data on cellular immunity were not available to reveal the full picture of immune response.

While our study provided solid data on cross-immunity among closely related coronaviruses, the findings should be interpreted with caution. Patients infected with SARS-CoV-1 often have more systemic involvement and developed prominent viremia, which provides a strong stimulation to the immune system,^22,23^ whereas SARS-CoV-2 infections are often limited to the respiratory tract and viremia is rarely detected.^24,25^ Therefore, whether natural infection with SARS-CoV-2 could confer a similarly long-lasting cross-clade immunity remains to be verified.

## Conclusions

The findings of this prospective cohort study suggest that infection with SARS-CoV-1 was associated with detectable levels of antibodies that cross-react and cross-neutralize SARS-CoV-2. These findings support the development of broadly protective vaccines to cover sarbecoviruses that caused 2 devastating zoonotic outbreaks in humans over the last 2 decades.

## Notes

Supplement.
Data Sharing Statement
