# Supplementary material for: Cross-Clade Memory Immunity in Adults Following SARS-CoV-1 Infection in 2003
Source: JAMA Netw Open. 2022 Dec 20;5(12):e2247723. doi: 10.1001/jamanetworkopen.2022.47723 (PMC9856533; doi:10.1001/jamanetworkopen.2022.47723)
Supplement: Supplement. — Data Sharing Statement [file jamanetwopen-e2247723-s001.pdf]

Data Sharing Statement

Ng. Cross-Clade Memory Immunity in Adults Following SARS-CoV-1 Infection in 2003. *JAMA Netw Open*. Published December 20, 2022. doi:10.1001/jamanetworkopen.2022.47723

Data
Data available: Yes
Data types: Deidentified participant data
How to access data: Please send request to corresponding author paulkschan@cuhk.edu.hk
When available: With publication

Supporting Documents
Document types: None

Additional Information
Who can access the data: Researchers whose proposed use of the data has been approved
Types of analyses: Vaccine response study
Mechanisms of data availability: After approval of a proposal and with a signed data access agreement
